# The effect of intracerebroventricular injection of insulin-like growth factor-1 on morphine-induced conditioned place preference extinction and reinstatement; a behavioral and biochemical experimental study

**DOI:** 10.1186/s12993-025-00304-y

**Published:** 2025-11-12

**Authors:** Erfan Ghadirzadeh, Mobina Gheibi, Ali Siahposht-Khachaki, Ehsan Vahdati Helan, Mohammad Farvardin, Shiva Shadi, Ali Abdolkarimi

**Affiliations:** 1 Student Research Committee, School of Medicine, Mazandaran University of Medical Sciences, Sari, Iran; 2 Non-Communicable Diseases Institute, Mazandaran University of Medical Sciences, Sari, Iran; 3 Student Research Committee, School of Allied Medical Sciences, Mazandaran University of Medical Sciences, Sari, Iran; 4 Immunogenetics Research Center, Department of Physiology, Mazandaran University of Medical Sciences, Sari, Iran; 5 Department of Internal Medicine, Faculty of Medicine, Tabriz University of Medical Sciences, Tabriz, Iran; 6 School of Medicine, Mazandaran University of Medical Sciences, Ramsar Campus, Sari, Iran

**Keywords:** Rats, Morphine, Conditioned place preference, Insulin-like growth factor-1, c-Fos

## Abstract

**Background:**

Morphine addiction is a growing problem with severe consequences. Interestingly, Insulin-like Growth Factor-1 (IGF-1), a hormone with the ability to modulate neural pathways and exert neuroprotective and regenerative properties, could emerge as a potential treatment. However, to the best of our knowledge, the role of IGF-1 in the extinction and reinstatement phases of morphine induced conditioned place preference (CPP) remains unexplored. Thus, this study aimed to investigate the behavioral and biochemical effects of intracerebroventricular (ICV) IGF-1 administration on extinction and reinstatement after morphine induced CPP and c-Fos expression in nucleus accumbens (NAc).

**Methods:**

Rats were conditioned with morphine (5 mg/kg, subcutaneously). The study examined alterations in CPP scores after administering varying multiple doses of IGF-1 (5, 10, and 20 µg) daily during the extinction and reinstatement phases of CPP, or single 20 µg dose administration prior to the extinction or prior to the reinstatement phase. Following these procedures, c-Fos levels in the NAc were quantified using the ELISA method.

**Results:**

The findings revealed that daily administration of IGF-1 at doses of 5, 10, and 20 µg resulted in a dose-dependent reduction in conditioning scores and shorter extinction period. Importantly, only the 20 µg attenuated morphine reinstatement significantly. Additionally, c-Fos levels, which increased following morphine exposure, were markedly reduced by IGF-1 administration across all phases.

**Conclusion:**

This study demonstrates that IGF-1 administration could facilitates the extinction and attenuate the reinstatement of morphine-induced CPP, highlighting its potential as a therapeutic strategy in opioid addiction.

## Background

Morphine addiction is a significant global health issue, driven by its widespread use in pain management and potential for misuse [1]. It presents substantial challenges, including severe health consequences, social and economic burdens, and treatment difficulties due to its highly addictive nature and the risk of relapse [2]. According to the World Health Organization (WHO), an estimated 15.5 million people worldwide suffer from opioid use disorders [3]. In the United States alone, the National Institute on Drug Abuse (NIDA) reports that over 2.7 million people aged 12 and older had an opioid use disorder in 2020, underscoring the pervasive societal impact of this crisis [4].

Addiction is traditionally characterized by compulsive drug use despite adverse consequences, often linked to physical and psychological dependence. It is also increasingly recognized as a disorder of learning and memory, involving disruptions in both instrumental and Pavlovian conditioning mechanisms [5, 6]. In the context of drug addiction, Pavlovian conditioning leads to the association of environmental cues, such as specific places or objects, with the drug’s rewarding effects, while instrumental conditioning drives the learned behaviors necessary to obtain the drug [7]. These maladaptive learning processes contribute to the persistence of drug-seeking behavior, as drug-associated cues become powerful triggers for craving and relapse, even after periods of abstinence.

Physiologically, addiction manifests as acquired tolerance, a diminished response to a drug after repeated exposure, requiring escalating doses to achieve the same effect, coupled with withdrawal symptoms upon cessation [8]. However, this view is incomplete without considering addiction as a disorder of maladaptive learning and memory, where drug-associated cues and contexts become entrenched triggers for craving and relapse [5, 9].

Studies have shown that drugs like morphine alter the brain’s reward system, particularly the mesolimbic dopamine pathway involving the ventral tegmental area (VTA), nucleus accumbens (NAc) [10], and prefrontal cortex [11], reinforcing drug-seeking through supraphysiological dopamine release [12]. The dopaminergic system, particularly the mesolimbic dopamine pathway, is critical for reinforcement learning, modulating motivation and behavioral responses to both rewarding and aversive stimuli [13, 14]. These drug-induced neuroadaptations create persistent memories that are resistant to erasure, contributing to the chronicity of addiction and relapse [15]. The learning processes in addiction are exemplified by extinction and reinstatement. Extinction involves new learning that competes with drug-associated memories, reducing conditioned responses to drug cues without eliminating the original memory [16]. Reinstatement, conversely, models relapse, where exposure to drug-related stimuli reactivates drug-seeking behavior even after abstinence [17]. These phases underscore the importance of targeting neuroplasticity and memory mechanisms for effective addiction therapies. Despite advances in understanding addiction’s neurobiology, current treatments, such as dopamine antagonists, often fail to address these underlying cognitive changes, limiting their efficacy [18].

Insulin-like growth factor 1 (IGF-1) has emerged as a promising therapeutic candidate due to its neuroprotective and restorative properties. IGF-1 is a 70-amino acid peptide primarily secreted by the liver but also synthesized in the central nervous system (CNS), particularly in the cerebral cortex, hippocampus, cerebellum, and hypothalamus [19]. It plays a critical role in neuronal development, synaptic plasticity, and neurogenesis [20]. In the developing brain, IGF-1 promotes neuronal proliferation, axonal growth, and synapse formation. In adults, it modulates synaptic plasticity, enhances neurogenesis, and supports mitochondrial function, which is essential for cognitive processes [21]. IGF-1 deficiency is associated with growth failure, microcephaly, and intellectual disability, highlighting its importance in brain function [22].

IGF-1 also exhibits potent neuroprotective effects, mitigating oxidative stress, inflammation, and neuronal damage [23]. It has been shown to enhance the survival of dopaminergic neurons, which are critical for reward processing and addiction [24]. Notably, IGF-1 administration prevents morphine-induced amnesia and improves cognitive function, suggesting its potential to counteract the neurological deficits associated with opioid addiction [25]. Furthermore, midbrain dopamine neurons synthesize and release IGF-1 in an activity-dependent manner, modulating dopamine synthesis and neuronal activity [24]. This neuromodulatory role positions IGF-1 as a key regulator of dopamine transmission and reward-related behaviors.

Despite these promising findings, to the best of our knowledge, the role of IGF-1 in the extinction and reinstatement phases of morphine conditioning remains unexplored. Thus, this study aims to investigate the behavioral and biochemical effects of intracerebroventricular (ICV) IGF-1 administration on extinction and reinstatement after morphine induced conditioned place preference (CPP).

## Materials and methods

### Animal preparation and study groups

In this study, 64 adult male Albino-Wistar rats (weighing 250–330 gr) were obtained from the Animal Institute of Mazandaran University of Medical Sciences (approved by the Laboratory Animals Research Ethics Committee at Mazandaran University of Medical Sciences with Ethics Approval Code: IR.MAZUMS.AEC.1402.014), with eight animals assigned to each six groups, and were kept in an animal house with 12:12 dark: light cycle, temperature 22 ± 2 °C, humidity 60–65%, and free food and water accessibility.

Animals were excluded if any of the following criteria were fulfilled during the study:

- Preconditioning (Pre-Test) Phase: Rats were excluded if they showed a strong pre-existing bias for one of the two side chambers (Chamber A or B) during the preconditioning phase. This bias was defined as spending time outside a calculated range in one chamber based on a 10-minute session.
- Post-Conditioning (Expression): Rats were excluded if they failed to demonstrate a significant preference for the morphine-paired chamber (Chamber A) compared to their pre-test behavior during the post-conditioning test.
- Cannula Placement Verification: Rats were excluded if post-mortem histological examination revealed incorrect cannula placement in the lateral ventricle, verified using the Rat Brain Paxinos and Watson atlas.
- Health and Locomotor Activity: Rats were excluded if they exhibited health issues or impaired locomotor activity that could hinder their ability to perform the task.

**Fig. 1:**
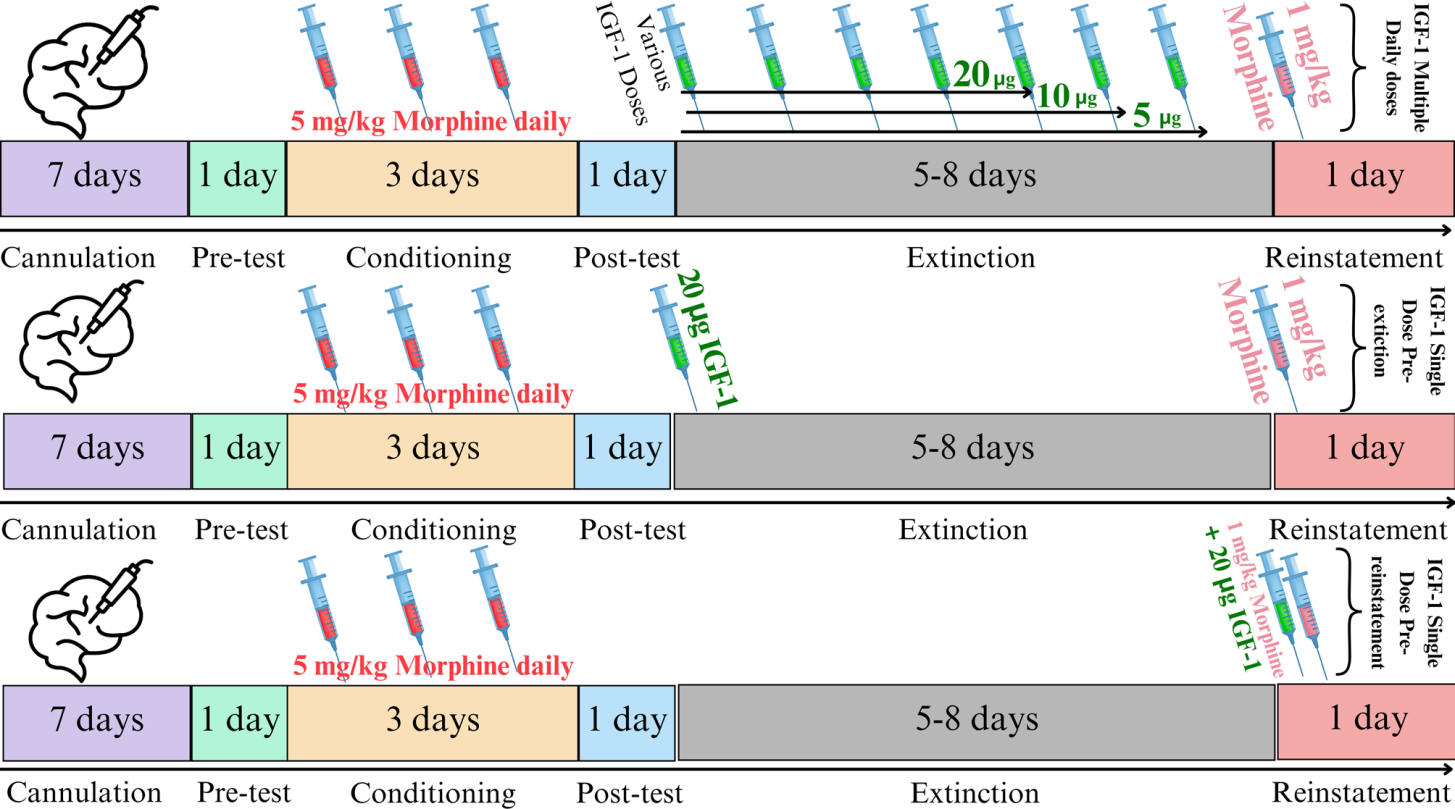
First Row: the experiment schedule for rats receiving repeated injections of IGF-1. Second Row: The experiment schedule for rats receiving single injections of IGF-1 pre-extinction. Third Row: The experiment schedule for rats receiving single injections of IGF-1 pre-reinstatement

The study comprised eight groups (eight rats each) structured as below:

1. Vehicle group (control): Rats were conditioned with subcutaneous (SC) injections of morphine sulfate (5 mg/kg, dissolved in 1 mL/kg normal saline [0.9% NaCl]) following the standard CPP protocol. During the extinction phase, these rats received daily ICV injections of 5 µL phosphate-buffered saline (PBS), the vehicle used for IGF-1. PBS was administered via a 5 µL Hamilton syringe through a cannula previously implanted in the lateral ventricle, one hour before each CPP test, until full extinction of the conditioned preference was achieved. To induce reinstatement, rats received a single SC injection of a low, non-conditioning dose of morphine (1 mg/kg, dissolved in 1 mL/kg normal saline) immediately before the reinstatement test. This group served as the control to evaluate the effects of morphine conditioning, extinction, and reinstatement in the absence of IGF-1 treatment.
2. Unpaired group Morphine without CPP (control): These rats only received SC injections of morphine sulfate (5 mg/kg, dissolved in 1 mL/kg normal saline [0.9% NaCl]) randomly without context association and place conditioning.
3. IGF-1 Conditioning Control Group: To address whether IGF-1 itself induces place conditioning, rats received ICV injections of IGF-1 at three doses (5 µg on first, 10 µg on second, and 20 µg on third days of conditioning, consecutively) instead of morphine during the conditioning phase. The conditioning protocol mirrored that of the morphine-conditioned groups.
4. Repeated IGF-1 Injection Groups (5, 10, and 20 µg): Identical to the vehicle control group, rats were conditioned with SC injections of morphine sulfate (5 mg/kg, dissolved in 1 mL/kg normal saline). During the extinction phase, rats received daily ICV injections of IGF-1 at doses of 5, 10, or 20 µg (dissolved in 5 µL PBS). Injections were administered one hour before each CPP test using a 5 µL Hamilton syringe through the implanted cannula, continuing until full extinction was achieved. Identical to the vehicle control group, rats received a single SC injection of morphine (1 mg/kg, dissolved in 1 mL/kg normal saline) to induce reinstatement. These three groups assessed the dose-dependent effects of repeated IGF-1 administration during the extinction phase on both the duration of extinction and the intensity of reinstatement (Fig. 1).

5. Single Dose IGF-1 Before Extinction Group: Rats were conditioned with SC injections of morphine sulfate (5 mg/kg, dissolved in 1 mL/kg normal saline). These rats received a single ICV injection of IGF-1 highest effective dose (20 µg dissolved in 5 µL PBS) one hour before the first CPP test of the extinction phase. No additional ICV injections were administered during the extinction period. Then, rats received a single SC injection of morphine (1 mg/kg, dissolved in 1 mL/kg normal saline) to induce reinstatement. This group evaluated the impact of a single IGF-1 dose at the onset of the extinction phase on extinction duration and subsequent reinstatement.
6. Single Dose IGF-1 Before Reinstatement Group: Rats were conditioned with SC injections of morphine sulfate (5 mg/kg, dissolved in 1 mL/kg normal saline). No ICV injections were administered during the extinction phase. These rats received a single ICV injection of IGF-1 highest effective dose (20 µg dissolved in 5 µL PBS) one hour before the reinstatement test, in addition to a single SC injection of morphine (1 mg/kg, dissolved in 1 mL/kg normal saline) to induce reinstatement. This group examined the effect of a single IGF-1 dose administered immediately prior to the reinstatement phase on the intensity of reinstatement.

All IGF-1 injections were conducted at 11 AM, a common practice to control for circadian rhythm effects in behavioral studies, ensuring consistency across experiments. The drug was infused over 60 s, consistent with standard intracranial injection protocols to ensure precise delivery and minimize tissue damage. A 1-hour delay was implemented between injection and the start of the conditioning session, allowing for adequate drug diffusion and action.

### Surgical procedure and cannulation

Before the pre-test, the animals underwent stereotaxic surgery for the implantation of a cannula into the lateral ventricle, guided by coordinates from the Paxinos and Watson Atlas [26]. First, anesthesia was induced through an intraperitoneal injection of a mixture containing 2% xylazine (10 mg/kg) and 10% ketamine (100 mg/kg). Then, using a Stoelting stereotaxic apparatus, an 8 mm long 30-gauge guide cannula, supported by a stainless steel screw and dental acrylic cement, was implanted into the lateral cerebral ventricle by following coordinates: anteroposterior = 0.5 mm caudal to bregma, lateral = 1.6 mm lateral to midline, dorsoventral = 4.2 mm ventral from the skull surface (guide cannula was implanted 1 mm above the appropriate injection place). Following the surgery, the animals were allowed a 7-day recovery period to ensure proper healing and stabilization before advancing to the conditioning protocol.

### Protocol for morphine-induced place conditioning

The experimental protocol was designed to investigate morphine-induced CPP, extinction, and reinstatement phases in an animal model [27]. The procedure consists of several phases, each with specific objectives and methodologies, as outlined below:

### CPP apparatus

The CPP paradigm is a well-established behavioral tool that specifically evaluates the Pavlovian conditioning component of drug reward by assessing an animal’s preference for an environment previously paired with drug administration [28]. In CPP, animals learn to associate a specific context with the rewarding effects of a drug, such as morphine, and subsequently exhibit a preference for that context over a neutral one. This makes CPP particularly suitable for studying the extinction phase, where repeated exposure to the drug-paired environment without the drug leads to a gradual reduction in preference, reflecting the unlearning of the drug-context association [29]. This extinction phase is critical for understanding how to weaken the conditioned responses that contribute to relapse in addiction [17]. Furthermore, CPP allows for the assessment of reinstatement, a model of relapse, where exposure to drug-related stimuli reactivates drug-seeking behavior even after extinction [15]. By measuring the time course of extinction and the potential for reinstatement, CPP provides valuable insights into the persistence of drug-associated memories and the efficacy of interventions aimed at reducing drug-seeking behavior.

The apparatus used was a three-chambered box (Borj Sanat, Iran), with two (A and B) equally sized side chambers (40 × 30 × 30 cm) differing only in visual cues and floor and wall contexture, and a smaller central chamber (30 × 15 × 40 cm) connecting the two. One compartment featured a grid floor, while the other had a perforated floor. The compartments were visually distinguished by wall patterns, stripes in one compartment and dots in the other, offering additional visual cues to reinforce contextual differentiation.

The connecting pathways between the central and side chambers were equipped with sliding doors. A video camera (Ethovision software) mounted above the CPP box continuously monitored and recorded the rats’ behavior, including time spent in each chamber and motor activity, which were later analyzed to calculate conditioning score (CPP score).

The CPP score = the time spent in the drug-paired compartment - the time spent in saline-paired compartment.

### Preconditioning (pre-test) phase (day 1)

The protocol begins with a pre-test phase to assess the animal’s baseline preference for either of the two distinct chambers (Chamber A or Chamber B) in the CPP apparatus. The animal was placed in the apparatus, which includes a neutral central corridor connecting the two chambers, and allowed to freely explore for 10 min. Animals exhibiting a strong bias, such as spending more or less time than the calculated time frame (Time frame: X ± 0.2X, X = 0.5 × (10 – time spend in central chamber) in one of the A or B chambers, were excluded from the study to ensure no pre-existing chamber preference influencing the results.

### Conditioning (acquisition) phase (day 2–4)

This study employed an unbiased conditioning approach, as recommended by Cunningham et al. [30], to mitigate any potential bias inherent in the apparatus design. Animals were randomly assigned to receive either the drug or vehicle in one of the two compartments, with assignments counterbalanced across groups. This randomization ensures that half the animals received the drug in the grid-floor/striped/bright compartment, while the other half received it in the hole-floor/dotted/dim compartment (and vice versa for the vehicle). To prevent misunderstanding, we name the chamber in which the animals receive morphine as chamber A, and the chamber in which animals receive saline as chamber B.

The conditioning phase spanned three days and was designed to establish a CPP for the chamber paired with morphine administration. On Day 1, half animals received a SC injection of morphine sulfate (5 mg/kg, an effective dose for conditioning) in the morning and were confined to Chamber A for 45 min. Six hours later (in the afternoon), the animals received injection of 1 mg/kg normal saline and were confined to Chamber B for 45 min. The remaining rats received saline first and then morphine in the same manner.

**Fig. 2:**
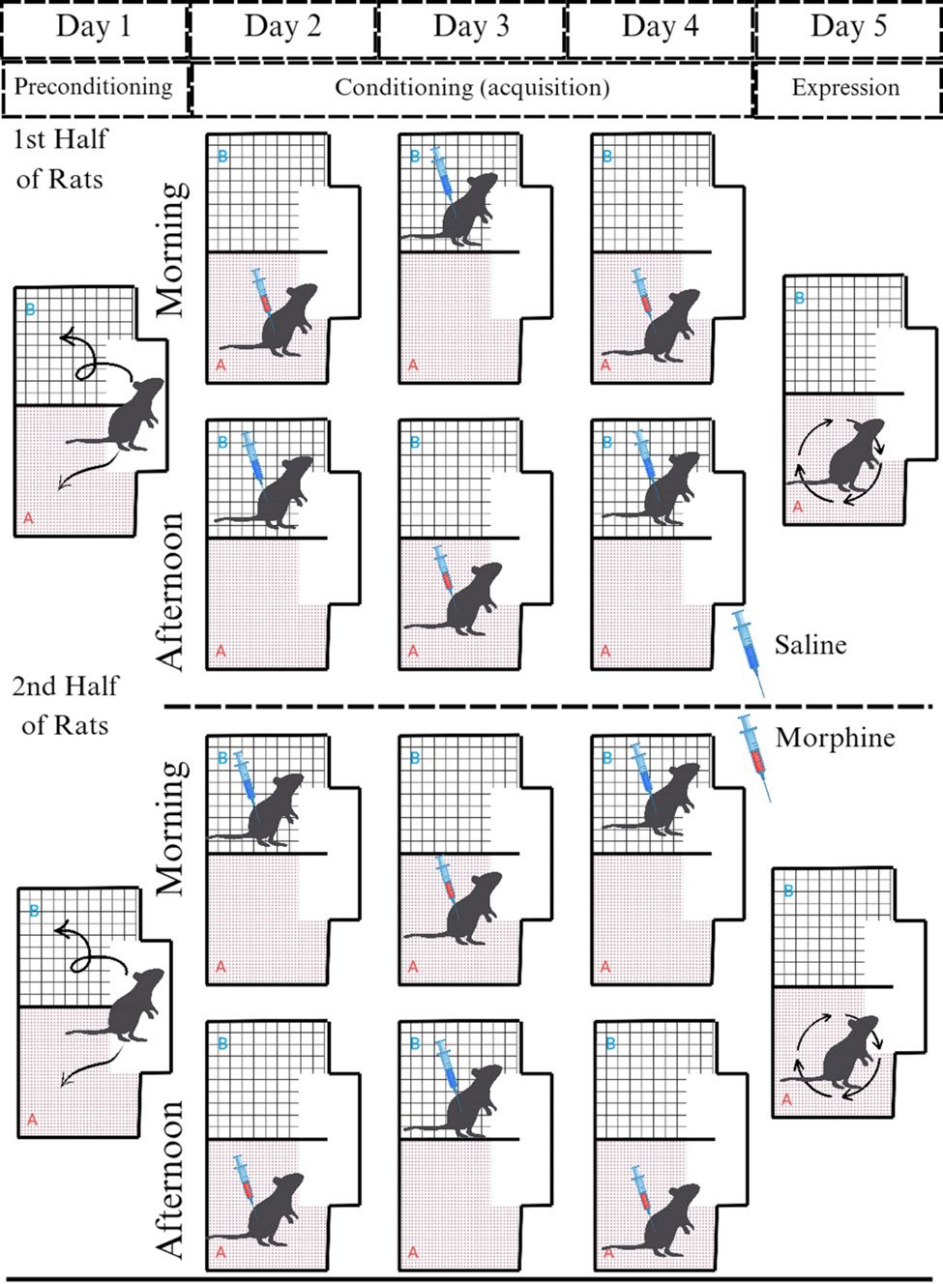
Graphical summary illustrating the induction of CPP through morphine sulfate (5 mg/kg) injections in rats. This study employed an unbiased conditioning approach. This illustration represent the half of the animals that received the morphine in the hole-floor/dotted/dim compartment. The other half of the rats (which are not shown in this illustration) received morphine in the grid-floor/striped/bright chamber in the same manner

On Day 2, the protocol was adjusted to prevent time-based conditioning. In the morning, the half that received morphine first on previous day, received saline and were confined to Chamber B for 45 min. After removal from Chamber B. Six hours later (in the afternoon), the animals received morphine and were confined to Chamber A for 45 min. The remaining half of the rats received morphine first and then saline in the same manner. On Day 3, the protocol mirrors that of Day 1 to reinforce the conditioning (Fig. 2).

### Post-conditioning (expression) test (post-test) (day 5)

After the three-day conditioning phase, a post-conditioning test was conducted to evaluate the success of the conditioning procedure. The animal was placed in the CPP apparatus and allowed to freely explore all chambers for 10 min. The time spent in each chamber was recorded, and successful conditioning was confirmed if the animal spent significantly more time in the morphine-paired chamber (Chamber A) compared to the pre-test phase. Animals failing to demonstrate this preference were excluded from the study.

### Extinction phase (day 6–12)

The extinction phase, which usually lasts about eight days according to previous studies [31], aimed to measure the time needed to gradually diminish the morphine induced CPP. During this phase, animals underwent daily CPP tests for 10 min. Prior to each test, the animal received an ICV injection of either IGF-1 (in different dose groups) or buffer phosphate (vehicle) as a control with no morphine injections. The extinction phase was considered complete when the animal no longer showed a preference for Chamber A, as indicated by the CPP scores returning to baseline levels observed during the pre-test phase for two consecutive days.

In this study, a choice-extinction procedure was employed, wherein animals were allowed to freely explore the entire CPP apparatus, configured identically to the test phase. This method enables animals to learn that the previously drug-paired compartment no longer predicts the drug effect, thereby facilitating the extinction of the conditioned preference. This approach contrasts with a forced-extinction procedure, where animals are confined to specific compartments, typically the drug-paired one, during extinction training to extinguish the association.

### Reinstatement phase (day 13)

The final phase of the protocol assesses relapse-like behavior by reinstating the conditioned preference after a drug withdrawal period (extinction). Animals received a SC injection of a low, non-conditioning dose of morphine (1 mg/kg), which is insufficient to induce conditioning but enough to trigger relapse due to prior sensitization. The animals were then placed in the CPP apparatus, and the time spent in each chamber with CCP scores were recorded. A significant increase in time spent in Chamber A indicated reinstatement of the conditioned preference.

### Verification of cannula placement

Upon completing the behavioral assessments, including extinction and reinstatement tests, the rats were placed under deep anesthesia and were then transcardially perfused with a 10% formalin solution and normal saline. To confirm the placement of the cannula, 50 μm coronal brain sections were extracted, fixed, and prepared. The exact location of the cannula was verified using the Rat Brain Paxinos and Watson atlas. Only subjects with accurately positioned cannulas were considered for data analysis.

### c-Fos assessment

The c-Fos protein is a well-established marker of neuronal activity, reflecting recent neuronal activation in response to a wide range of stimuli, including but not limited to reward-related processes, stress, and aversive events. Previous studies have demonstrated that morphine administration significantly elevates c-Fos levels in the NAc, correlating with drug-seeking behavior [32]. In the context of this study, c-Fos expression in the NAc was used to assess neuronal activation following morphine exposure and IGF-1 treatment, given the NAc’s critical role in reward processing and addiction.

Following the reinstatement phase, the rats were euthanized, and their brains were promptly extracted. The brain was positioned in a stainless steel brain matrix, allowing for the bilateral dissection of the NAc based on stereotaxic coordinates of Paxinos & Watson atlas. The reference points used for dissection were anteroposterior 2.04 mm, mediolateral 1.6 mm, and dorsoventral 7 mm from the bregma. c-Fos protein levels in the NAc were measured using the Enzyme-linked immunosorbent assay (ELISA) method using rat c-Fos ELISA kits (MBS005753) from Mybiosource, Inc. (San Diego, CA, USA).

### Statistical analysis

For data analysis, the normality of data distribution was first assessed using Shapiro-Wilk test. One-way ANOVA was used to compare quantitative variables between the tested groups followed by Newman-Keuls post hoc test in case of normal distribution. For two-way ANOVA, Tukey’s post hoc test was used when required. In cases of non-normal distribution data, the Kruskal-Wallis test was employed followed by Dunn’s multiple comparison test. Also, unpaired t-test was applied to determine the significance of difference between two groups. A significance level of 0.05 (*P* ≤ 0.05) was considered for all analyses. Statistical analyses were performed using GraphPad Prism 8 software, and all data were expressed as Mean ± SEM.

## Results

### Locomotor activity

**Fig. 3:**
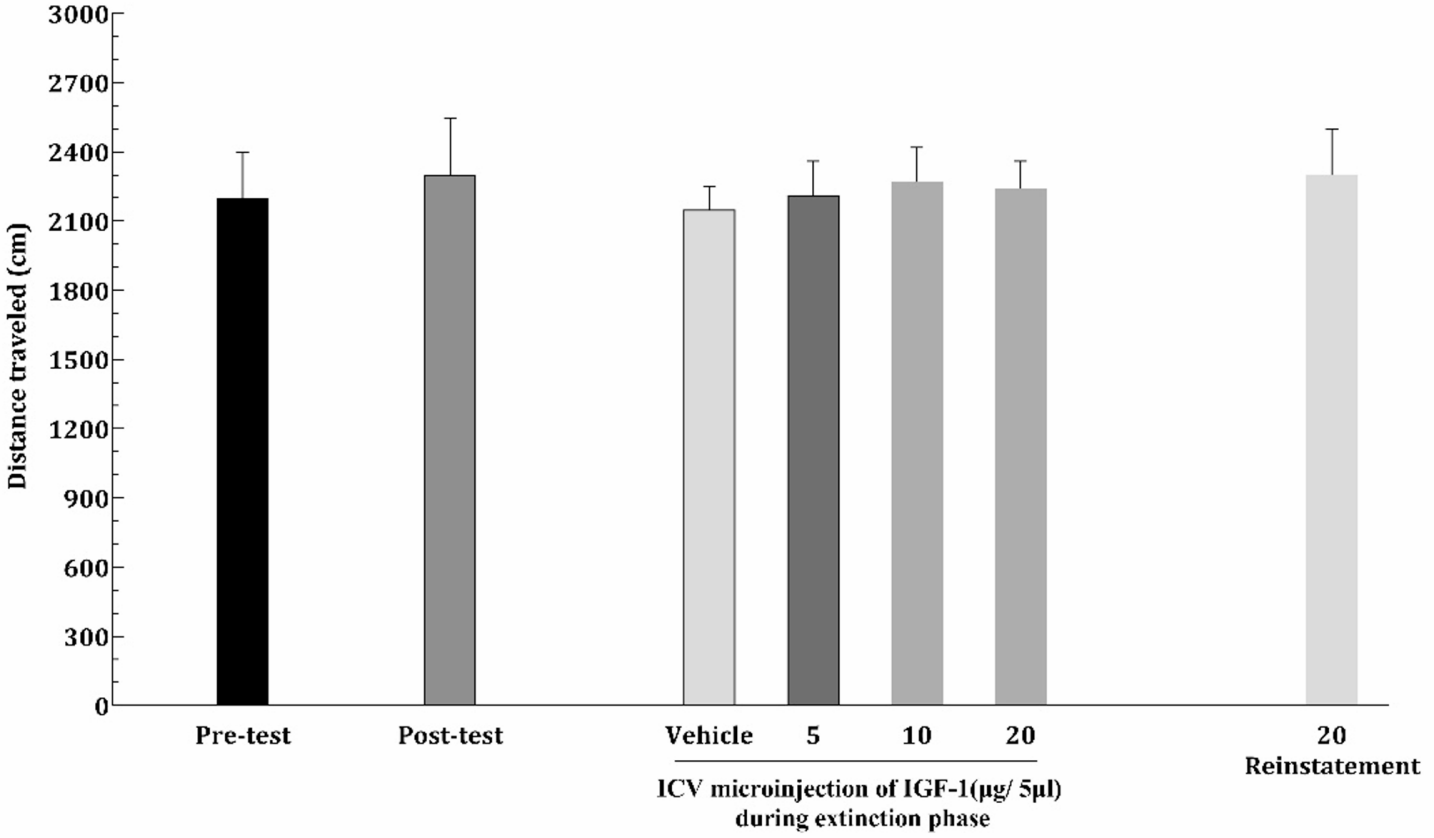
The effect of ICV daily administration of morphine solvent or different IGF-1 daily doses on the locomotor activity of male rats. Distance traveled by all these animals were almost equal in all phases. Data are shown as mean ± SEM for eight rats

We assessed locomotor activity to ensure that all rats were in optimal health and capable of unrestricted movement. This evaluation confirmed that any preference for a specific chamber was attributable to the rats’ inherent preferences rather than any physical incapacity or as a result of morphine or IGF-1 effects of the animals’ motor function. As illustrated in Fig. 3, all rats across the experimental groups demonstrated sufficient mobility to navigate the apparatus effectively. No significant differences in locomotor efficiency were observed among the groups, indicating consistent and unimpaired movement throughout the study. Furthermore, the administration of varying doses of IGF-1 and morphine at different phases did not alter motor activity, ensuring that the calculation of the CPP score remained unaffected by locomotor influences (F[6,49]: 0.1476, *P* = 0.9601).

### Daily repeated ICV administration of solvent (vehicle) or IGF-1 during the extinction period

**Fig. 4:**
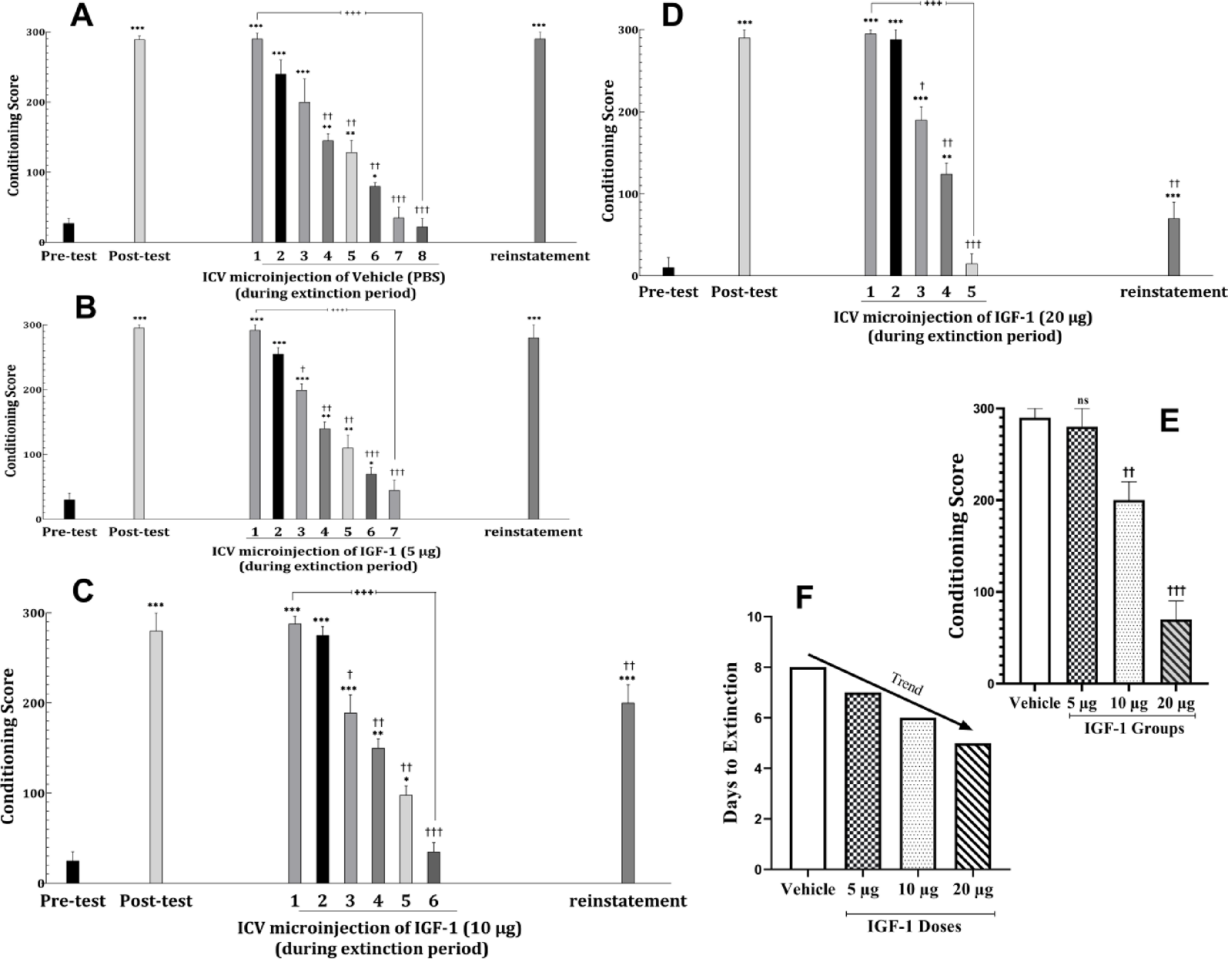
The effect of daily ICV drug administration during the extinction period on the duration of extinction period and reinstatement intensity of morphine-induced CPP in male rats. Animals received medication, one hour before the CCP test on all days of the extinction period (**A**: Vehicle, **B**: 5 µg IGF-1, **C**: 10 µg IGF-1, and **D**: 20 µg IGF-1). Data are shown as Mean ± SEM for eight rats. **E**: direct comparison of conditioning scores in reinstatement phase between vehicle group and each IGF-1 dosage groups, showing significant reductions in 10 and 20 µg doses. **F**: direct comparison of elapsed days until induction of extinction between vehicle group and each IGF-1 dosage groups, showing a reduction trend with increase in IGF-1 dosage. *P < 0.05, **P < 0.01 and ***P < 0.001 different from the pre-test day. ^†^*P* < 0.05, ^††^*P* < 0.01, ^†††^*P* < 0.001 different from the post-test day. ^+++^*P* < 0.001

In the vehicle (solvent) control group, the extinction period lasted for eight days. The CPP score measured on the reinstatement day did not differ significantly from the CPP score on the first expression day or the post-test day, indicating that solvent had no effect on the reinstatement of morphine-induced CPP (Fig. 4A) (F[10, 76]: 11.8669, *P* < 0.001).

IGF-1 administration significantly influenced the extinction phase of CPP. There was a trend suggesting that higher doses of IGF-1 (5 µg, 10 µg, and 20 µg) may lead to greater reductions in the extinction period and reinstatement of CPP. Administration of 5 µg IGF-1 shortened the extinction period by 1 day compared to the control group. However, this dose did not alter the reinstatement phase, as the CPP score during the reinstatement phase remained comparable to that of the post-test day (Fig. 4B) (F[9,70]: 9.9806, *P* < 0.001).

At 10 µg, IGF-1 reduced the extinction period to six days, compared to eight days in the control group (F[8,63]: 11.6775, *P* < 0.001). This dose also partially attenuated the reinstatement phase, although the effect was less pronounced than with 20 µg (Fig. 4C). The 20 µg dose of IGF-1 produced the most significant effects (F[7,56]: 10.9808, *P* < 0.001). Rats treated with this dose exhibited a 3 day reduction in the extinction period compared to the control group. Additionally, the CPP score during the reinstatement phase was significantly lower than on the post-test day (*P* < 0.01), indicating that this dose effectively suppressed the reinstatement of morphine-induced CPP (Fig. 4D). Comparison of conditioning scores in the reinstatement phase shows significant reductions in IGF-1 10 and 20 µg doses (Fig. 4E). Also, as shown in Fig. 4F, days until reaching extinction shows a trend toward reduction with increasing IGF-1 dosage.

### ICV administration of a single effective IGF-1 dose (20 µg) before the extinction period on the duration of extinction and the reinstatement intensity of morphine-induced CPP

**Fig. 5:**
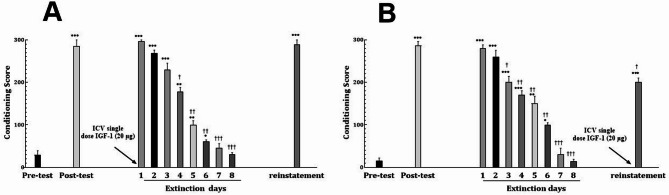
**A** The effect of single dose administration of IGF-1 pre-extinction on the duration of extinction period and reinstatement intensity of morphine-induced CPP in male rats. Animals received 20 µg/µL of IGF-1, after the post-conditioning phase one hour before the CPP test. **B**: The effect of single dose administration of IGF-1 pre-reinstatement on the reinstatement intensity of morphine-induced CPP in male rats. Animals received 20 µg/µL of IGF-1, prior to the reinstatement phase one hour before the CPP test. Data are shown as Mean ± SEM for eight rats. *P < 0.05, **P < 0.01 and ***P < 0.001 different from the pre-test day. ^†^*P* < 0.05, ^††^*P* < 0.01, ^†††^*P* < 0.001 different from the post-test day

The duration of the extinction period and the reinstatement intensity (F[10,76]: 17.8087, *P* < 0.001) of morphine-induced CPP were not significantly reduced when IGF-1 was administered as a single effective dose (20 µg ICV) prior to the initiation of the extinction phase (Fig. 5A).

### ICV administration of a single effective IGF-1 dose (20 µg) before the reinstatement period on the reinstatement intensity of morphine-induced CPP

Although a single ICV injection of IGF-1 (20 µg) administered prior to the initiation of the extinction phase did not lead to reduction of reinstatement, when given prior to the beginning of the reinstatement phase, it significantly decreased reinstatement intensity of morphine-induced CPP (Fig. 5B) (F[10,76]: 13.9098, *P* < 0.001).

### The effect of ICV administration of IGF-1 on c-Fos levels in the NAc

**Fig. 6:**
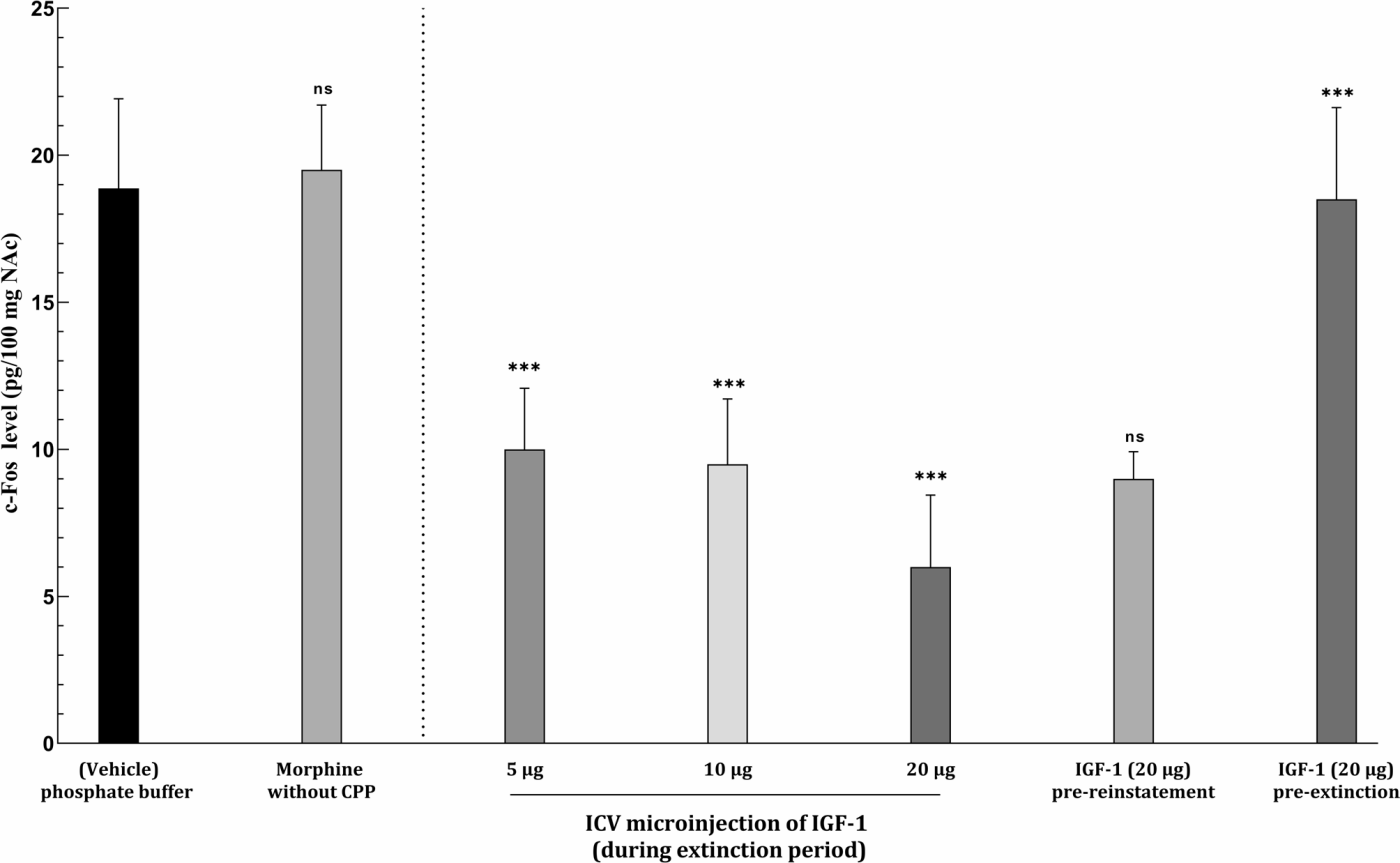
Evaluation of c-fos level in the NAc following ICV injection of various IGF-1 doses. Data are shown as Mean ± SEM for eight rats. ***P < 0.001 different from vehicle and “ns” as not significant (*P* > 0.05)

Morphine administration significantly elevated c-Fos levels in the NAc in the solvent control group. However, ICV administration of IGF-1 at various stages of the experiment led to a notable reduction in c-Fos protein levels (F[6, 49]: 39.763, *P* < 0.001). This effect was dose-dependent, with higher doses of IGF-1 producing a more pronounced decrease in c-Fos expression (Fig. 6).

### IGF-1 conditioning control group

**Fig. 7:**
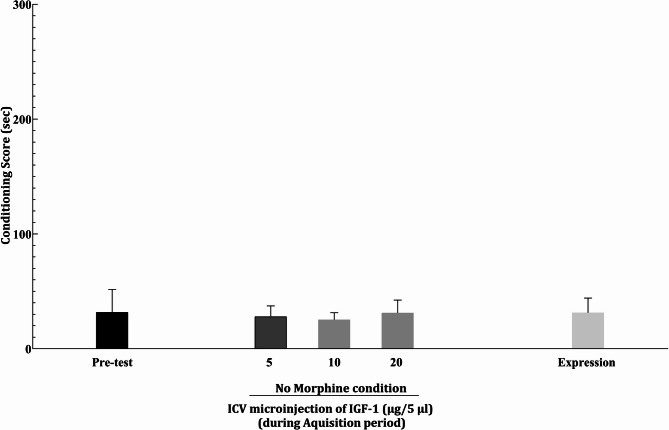
The effect of IGF-1 administration (without morphine conditioning) on CPP score. Animals received 5, 10, and 20 µg of IGF-1 in days 2, 3, and 4 of the conditioning phase, respectively

As shown in Fig. 7, the post-conditioning test showed no significant difference in the CPP score from pre-test (*P* > 0.05).

## Discussion

Our study aimed to evaluate the efficacy of ICV administration of IGF-1 in morphine-conditioned rats across different phases of the CPP paradigm, including, extinction, and reinstatement. We found that daily repeated ICV injections of IGF-1 (5, 10, and 20 µg) during the extinction phase reduced the conditioning score, shortened the duration of extinction, and attenuated the intensity of reinstatement, with the most pronounced effects observed at the highest dose (20 µg), suggesting a possible dose-dependent effect. However, the dose-dependent nature of IGF-1’s effects should be interpreted with caution pending further analysis.

IGF-1 is a polypeptide hormone produced locally in neurons and glial cells within the brain, as well as derived from the peripheral circulation [33]. It binds to the IGF-1 receptor (IGF-1R) and activates downstream signaling pathways, such as phosphoinositide 3-kinase (PI3K)-AKT and rat sarcoma virus-mitogen-activated protein kinase (RAS-MAPK) [34], which are critical for regulating neuronal survival, synaptic plasticity, and cognitive function [35].

These pathways directly influence neuronal excitability and synaptic transmission, processes that are often dysregulated in addiction [36]. In our study, IGF-1 administration during the extinction phase shortened the extinction period and reduced reinstatement, suggesting that it enhances the brain’s ability to “unlearn” drug-associated behaviors and resist relapse. This aligns with IGF-1’s role in restoring synaptic plasticity and reversing the neural changes induced by chronic morphine use.

Our findings align with previous research highlighting the role of the dopaminergic system in morphine addiction. Beitner-Johnson and Nestler [37] demonstrated that chronic morphine exposure decreases tyrosine hydroxylase (TH) levels in the VTA, a critical region in the brain’s reward circuitry. This reduction in TH, the rate-limiting enzyme in dopamine synthesis, suggests a downregulation of dopamine production, which may contribute to the development of tolerance and dependence. In contrast, Hashiguchi et al. [38] found that IGF-1 enhances TH activation in bovine chromaffin cells, indicating a potential role for IGF-1 in upregulating catecholamine synthesis. Although their study focused on peripheral cells, it raises the possibility that IGF-1 could similarly influence TH activity in central dopaminergic neurons.

Extending these insights, Dahmer et al. [39] showed that IGF-1 further enhances TH activation in bovine chromaffin cells, increasing catecholamine synthesis and supporting IGF-1’s capacity to normalize dopamine dynamics. In our study, the administration of IGF-1 during the extinction phase of morphine-induced CPP led to a dose-dependent reduction in conditioning scores and a shorter extinction period. This could suggest that IGF-1 may counteract the morphine-induced downregulation of TH, thereby restoring dopaminergic function and facilitating the extinction of drug-seeking behavior. Collectively, these studies support the hypothesis that IGF-1 modulates the dopaminergic system and synaptic plasticity, both of which are central to addiction and the extinction of drug-seeking behaviors. The reduction in NAc c-Fos expression in our results further corroborates this, as diminished neuronal activation in dopamine-recipient regions like the NAc likely underlies the observed decrease in CPP reinstatement.

Building on this dopaminergic framework, IGF-1’s neuroprotective effects may also explain its ability to accelerate extinction and prevent relapse in our model. Park et al. [40] demonstrated that central IGF-1 administration decreases depressive-like behavior and pro-inflammatory cytokine expression (e.g., IL-1β, TNF-α) while increasing brain-derived neurotrophic factor (BDNF) in mice. These findings are particularly relevant to our study, as chronic morphine exposure often induces neuroinflammation and emotional deficits that perpetuate addiction and hinder extinction learning.

By reducing inflammation and boosting BDNF (key drivers of synaptic remodeling), IGF-1 likely mitigates these effects, facilitating faster unlearning of morphine-associated memories and reducing reinstatement vulnerability, as observed in our results. Similarly, Naylander et al. [41] showed that growth hormone (GH) protects primary cortical cells against methadone-induced toxicity, while both GH and IGF-1 promote recovery of mitochondrial function and membrane integrity in cells pre-treated with methadone. This restorative mechanism provides a cellular basis for our behavioral results, where IGF-1 (especially at higher doses) repairs morphine-induced neuronal damage in reward circuits like the NAc, enabling quicker extinction and blunted reinstatement responses. The consistent reduction in c-Fos levels across doses in our study supports this neuroprotection, indicating stabilized neuronal activity post-morphine challenge.

Furthermore, IGF-1’s procognitive properties may underpin its enhancement of extinction learning. Brolin et al. [42] reported that chronic morphine administration impairs spatial memory in rats, correlating with reduced GluN2b expression in the frontal cortex. This suggests that morphine disrupts glutamatergic transmission, which is critical for learning and memory, processes also involved in extinguishing drug-related associations. Given IGF-1’s established role in enhancing synaptic plasticity and cognition, our finding that IGF-1 accelerates CPP extinction may reflect its ability to restore glutamatergic function and prefrontal-NAc connectivity. This enables rats to more effectively “unlearn” the morphine-place association, consistent with the shorter extinction durations and attenuated reinstatement we observed, particularly with repeated dosing.

Hashiguchi et al. [38] showed that acute central morphine administration increases GH and IGF-1 levels transiently in rats, suggesting opiates stimulate the GH/IGF-1 axis short-term. Conversely, Beitner-Johnson et al. [43] found that chronic morphine decreases IGF-1 levels in the VTA and plasma, possibly via HPA axis activation and elevated glucocorticoids, which inhibit IGF-1 production. This chronic reduction may contribute to addiction-related neuroadaptations, such as reduced neurofilament proteins in the VTA. In our study, exogenous IGF-1 administration likely counteracts this depletion, replenishing IGF-1 levels to mitigate morphine’s effects. The dose-dependent reduction in CPP scores and attenuated reinstatement support this, as IGF-1 may restore neuronal function impaired by chronic morphine exposure.

One of the key mechanisms by which IGF-1 exerts its effects is through the PI3K/Akt/mTOR pathway, which regulates protein synthesis and synaptic remodeling [44]. Activation of this pathway enhances the expression of synaptic proteins like PSD-95 and synapsin 1, which are critical for maintaining synaptic efficacy [45]. In models of opioid addiction, IGF-1 has been shown to restore Akt and S6 signaling in the prefrontal cortex, which are downregulated after prolonged opioid cessation. This restoration of signaling pathways likely contributes to the observed reduction in drug-seeking behavior [46]. In our study, not only did multiple daily IGF-1 doses reduce the extinction period and reinstatement intensity, but also even a single dose of IGF-1 given before the reinstatement period reduced the relapse and drug-seeking behavior.

Additionally, IGF-1 acts as a potent neuroprotective agent, shielding neurons from damage caused by oxidative stress, inflammation, and excitotoxicity. It activates survival pathways, such as the PI3K/Akt pathway, which inhibit apoptosis and promote cell resilience [47]. In our study, the highest dose of IGF-1 (20 µg) not only shortened the extinction period but also significantly reduced the reinstatement of morphine-induced CPP, indicating its potential to prevent relapse. This neuroprotective effect may be due to IGF-1’s ability to counteract the neurotoxic effects of chronic morphine use.

Our findings suggest that IGF-1 modulates the dopaminergic system to facilitate extinction and attenuate reinstatement of morphine-induced CPP. Chronic morphine exposure dysregulates dopamine signaling, reducing TH levels in the VTA [37], while drug cues trigger exaggerated dopamine release in the NAc [48]. IGF-1, known to enhance TH activity [38], likely normalizes this imbalance rather than universally upregulating dopamine, reducing cue-induced hyperactivity. During extinction, the dopaminergic system signals negative prediction errors, decreases in dopamine release when the expected reward is absent, driving the learning that the drug-paired chamber no longer predicts morphine [49, 50]. IGF-1 may accelerate this process by stabilizing dopamine signaling or enhancing synaptic plasticity in the NAc, enabling faster unlearning of the conditioned association. Additionally, the reduction in NAc c-Fos expression post-reinstatement with IGF-1 treatment suggests diminished neuronal activation to relapse triggers, possibly via altered dopamine responses. These effects align with reports that IGF-1 in the prefrontal cortex attenuates drug-seeking [46], highlighting its potential to modulate reward circuits broadly.

In addition to behavioral outcomes, we evaluated the expression of c-Fos protein, a marker of neuronal activation, across different phases and doses of IGF-1 administration [51]. c-Fos expression provides insights into the neural circuits involved in morphine addiction and the effects of IGF-1 on these circuits [52]. Our preliminary data suggest that IGF-1 modulates c-Fos expression in the NAc, a brain region critical for reward processing and decision-making [53]. These findings support the hypothesis that IGF-1 exerts its therapeutic effects by modulating activity in addiction-related neural circuits.

### Limitations

While our study provides valuable insights into the potential of IGF-1 for treating morphine addiction, several limitations should be acknowledged. First, the mechanisms underlying IGF-1’s effects on morphine-induced CPP remain incompletely understood. Future studies should investigate the specific signaling pathways and neural circuits involved. Second, the long-term safety and efficacy of IGF-1 administration need to be evaluated in preclinical and clinical settings. Finally, the role of IGF-1 in combination with other therapies, such as cognitive-behavioral interventions, warrants further exploration.

The NAc was chosen because it is a central hub in the reward circuit and is critically involved in the processing of drug-related cues and the expression of conditioned responses. By focusing on the NAc, we aimed to assess how IGF-1 modulates neuronal activity in this key region during the extinction and reinstatement phases of morphine-induced CPP. While our study concentrated on the NAc, we acknowledge that other brain regions, such as the prefrontal cortex, amygdala, and hippocampus, are also involved in these processes. Future research should explore how IGF-1 affects neuronal activity in these areas to provide a more comprehensive understanding of its therapeutic potential.

## Conclusion

This study provides novel evidence that intracerebroventricular administration of IGF-1 significantly modulates critical phases of morphine addiction in a rat model of CPP. Our key findings demonstrate that repeated daily administration of IGF-1 during the extinction phase produces dose-dependent effects: higher doses (10 and 20 µg) accelerated the extinction of morphine-induced CPP, and critically, the 20 µg dose robustly attenuated reinstatement, a model of relapse. Importantly, a single dose of IGF-1 (20 µg) administered prior to the reinstatement phase also effectively reduced reinstatement intensity, highlighting its potential acute protective effect against relapse triggers. Biochemically, these behavioral improvements were strongly correlated with a dose-dependent reduction in c-Fos expression within the nucleus accumbens, a key hub of the brain’s reward circuitry. Future research should focus on exploring systemic delivery methods, elucidating the specific molecular pathways (e.g., PI3K/AKT, MAPK) and neuronal subpopulations within the NAc and broader reward circuitry modulated by IGF-1, and evaluating its long-term efficacy and safety in diverse models of addiction and relapse prevention.

## Data Availability

The data are available upon reasonable request from the corresponding author.
